# WORKING THE SCHOLAR-ACTIVIST HYPHEN: QUEER GERONTOLOGY PERSPECTIVES FROM OLDER BLACK LESBIANS

**DOI:** 10.1093/geroni/igae098.1945

**Published:** 2024-12-31

**Authors:** Austin Oswald

**Affiliations:** Dalhousie University, Nova Scotia, Canada

## Abstract

The LGBTQ+ aging population is growing increasingly racially and ethnically diverse; however, older adults of color are underrepresented in LGBTQ+ aging research. This significant gap in the field constitutes a critical ethical issue that future scholarship must address. Advancing LGBTQ+ aging research necessitates a heightened focus and specificity on subgroups, particularly those with intersecting marginalized identities, while generating knowledge that challenges structural oppression. This presentation sheds light on insights gleaned from an ongoing, multi-year participatory action research project aimed at addressing gaps within contemporary LGBTQ+ aging research by highlighting the needs and experiences of LGBTQ+ older adults of color within an age-friendly city. Five older Black lesbian women, aged between 65 and 75, actively participated in designing, implementing, and evaluating a research project that analyzed data from public records and focus groups. Findings emphasize three fundamental principles of queer gerontology, providing tangible examples of community-engaged scholarship that researchers can utilize to advance the field of LGBTQ+ aging. This includes strategies aimed at deepening visibility and awareness of underrepresented subgroups through an intersectional lens, applying critical perspectives to challenge dominant frameworks that narrowly define old age and aging through heterosexual and gender norms, and combating oppression and injustice through activist-scholarship committed to real-world change. The insights from this presentation underscore the imperative for inclusive, community-engaged scholarship to advance our understanding of LGBTQ+ aging, promote social justice, and effect meaningful change in the lives of older LGBTQ+ adults with multiple intersecting identities.

